# Properties and Applications of Intelligent Packaging Indicators for Food Spoilage

**DOI:** 10.3390/membranes12050477

**Published:** 2022-04-28

**Authors:** Yuchen Ma, Wei Yang, Yujie Xia, Wenshuang Xue, Haixia Wu, Zhanming Li, Fang Zhang, Bin Qiu, Caili Fu

**Affiliations:** 1 Food Science and Technology Center, National University of Singapore Suzhou Research Institute, Suzhou 215123, China; yuchen.ma@nusri.cn (Y.M.); yujie.xia@nusri.cn (Y.X.); wenshuang.xue@nusri.cn (W.X.); 2 College of Chemistry and Chemical Engineering, Inner Mongolia University, Hohhot 010021, China; 3 Quality and Technology Center, Hainan Xiangtai Fishery Co., Ltd., Haikou 571924, China; yw@esfish.com; 4 Engineering Research Center of Dairy Quality and Safety Control Technology, Ministry of Education, Inner Mongolia University, Hohhot 010021, China; 5 School of Grain Science and Technology, Jiangsu University of Science and Technology, Zhenjiang 212100, China; lizhanming@just.edu.cn; 6 College of Biological Science and Engineering, Fuzhou University, Fuzhou 350116, China; fangzh921@fzu.edu.cn; 7 Ministry of Education Key Laboratory for Analytical Science of Food Safety and Biology, Fujian Provincial Key Laboratory of Analysis and Detection for Food Safety, College of Chemistry, Fuzhou University, Fuzhou 350116, China; summer328cn@163.com

**Keywords:** food packaging, indicator, spoilage, 3D printing, color

## Abstract

Food packaging plays a vital role in the food supply chain by acting as an additional layer to protect against food contamination, but the main function of traditional conventional packaging is only to isolate food from the outside environment, and cannot provide related information about food spoilage. Intelligent packaging can feel, inspect, and record external or internal changes in food products to provide further information about food quality. Importantly, intelligent packaging indicators will account for a significant proportion of the food industry’s production, with promising application potential. In this review, we mainly summarize and review the upcoming progress in the classification, preparation, and application of food packaging indicators. Equally, the feasibility of 3D printing in the preparation of intelligent food packaging indicators is also discussed in detail, as well as the limitations and future directions of smart food packaging. Taken together, the information supported in this paper provides new insights into monitoring food spoilage and food quality.

## 1. Introduction

Food spoilage is not only associated with human health, but also economic losses for the food industry [1]. Approximately 1.3 billion tons of food are destroyed or lost annually during the food supply process [2]. The food shortage due to the recent new crown epidemic has, further, drawn great attention to food security. Food quality can be decreased by different agents (physical, chemical, and biological) in the process of food storage, transportation, and marketing, as well as microorganisms, enzymes, temperature, and other factors, leading to food spoilage [3]. Therefore, it is significant to establish a method for easy and real-time food quality and safety testing.

Food packaging is designed to guarantee the quality, health, integrity, and safety of food products, and is a system that coordinates the transportation, storage, and retail sale of food [4,5]. Food packaging plays a vital role in the food supply chain by acting as a protective layer against food pollution, but the main function of traditional conventional packaging is only to separate the food from the outside environment, and consequently, it cannot provide information about food spoilage [3]. Conventional methods for determining food spoilage, including physical and chemical testing, microbial characterization, and sensory evaluation, are inconvenient due to the disadvantages of cumbersome pre-treatments and operations, being time-consuming, and incurring high costs, which makes it difficult to achieve a non-destructive and rapid detection method for food quality [6]. Although the protective effect of food packaging can delay the spoilage of food to a certain extent, it is impossible to completely avoid the spoilage of food. Food undergoes many changes during the spoilage process, but it is difficult to assess in most cases, and intelligent packaging emerges as a present-day requirement. Intelligent packaging can sense, detect, and record changes on the outside or inside of products to deliver food safety and quality information to consumers [2].

**Figure 1:**
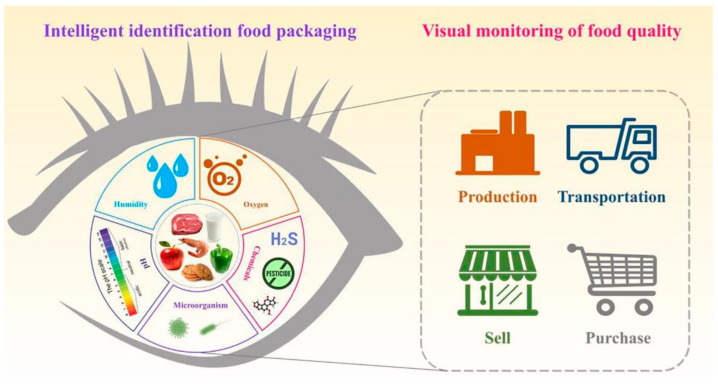
Intelligent food packaging monitors food quality. Reproduced from Ref. [8] with permission.

Food packaging containers and materials are divided into seven categories: plastic, paper, glass, ceramic, metal, composite packaging, and other packaging. Plastic packaging is widely used for tea, fruit juice, etc., due to its lightness, portability, and low cost. Paper packaging materials, with the characteristics of low price, good protection, and easy recycling, are used for cartons and paper bags. Glass packaging has good and reliable chemical stability and barrier properties, and its applications include grain and oil bottles, storage tanks, glass tableware, etc. Ceramic products are widely used in daily life and metal packaging materials can prevent food deliquescence, deterioration, spoilage, and changes in flavor. At present, metal food packaging materials are mainly used for canned meat and milk powder. Currently, in order to reduce unnecessary food waste and meet consumers’ needs for food safety and quality, intelligent food packaging has come into being. Generally, intelligent packaging can feel, inspect, and chart changes in the exterior or interior of food products, providing further information on food quality (Figure 1) [7]. Intelligent food packaging may realize the food processing process, from the supply of the raw materials to the products’ manufacturing, packaging, distribution, sale, packaging waste disposal, and other aspects of information, including sensing, storage, transmission, and feedback functions [4].

Intelligent packaging is currently divided into three main categories [3], including indicators (providing more convenience and food quality information for consumers), data carriers (storage, distribution, and traceability), and sensors (to quickly and efficiently measure food products). Intelligent packaging will occupy a large proportion of the food industry’s production in the future, with wide market prospects [8]. In this paper, we mainly summarize and outline the upcoming progress in the classification, preparation, and application of food packaging indicators. Equally, the feasibility of 3D printing for preparing intelligent food packaging indicators is also discussed, as are the limitations and future directions of intelligent food packaging.

## 2. Indicators’ Properties

As widely used intelligent food packaging, indicators are mainly applied to predict the shelf life of food products and to communicate food quality and safety information, as well as other characteristics, to consumers by the presence of certain chemical or biological substances inside the food packaging [3,4]. Indicators can be classified into various types, either for the interior or exterior of food, according to the package type [9].

### 2.1. Indicator Classification

The various indicators can be divided into time–temperature indicators (TTIs), freshness indicators (FIs), leak indicators (LIs), or pH indicators (PHIs), according to the mechanism of action and the applications, as shown in Table 1 [7,10,11,12,13,14,15,16,17,18,19,20,21,22,23,24].

#### 2.1.1. Time–Temperature Indicators (TTIs)

**Figure 2:**
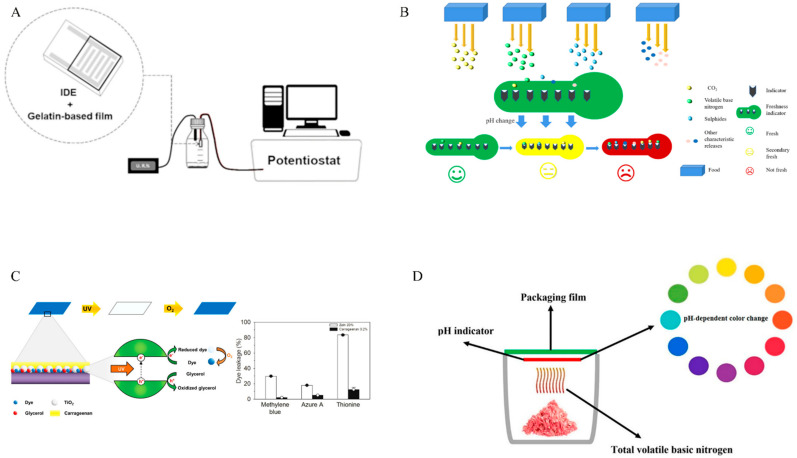
The working principle of indicators in food intelligent packaging. (**A**) TTI; (**B**) FI; (**C**) LI; (**D**) PHI. Reproduced from Refs. [2,25,26,27] with permission.

TTIs record the historical temperature changes of packaged food products during the packaging, storage, distribution, and retail periods [4]. Through this irreversible data, consumers and distributors are provided with food quality and safety information (Figure 2A). According to the mechanism of action, TTIs can be divided into diffusion types, microbial types, enzyme types, polymerization types, and light color types. These indicators are primarily applicable to temperature-sensitive foods, including frozen foods and chilled foods [6].

#### 2.1.2. Freshness Indicators (FI)

When fresh food products are packaged, the metabolites change constantly due to internal chemical changes, biological activities, and other factors (Figure 2B). The freshness indicator transmits quality information to consumers and distributors by detecting these metabolites or by making visible color changes with the metabolites [4,6]. Metabolites such as glucose, organic acids, ethanol, volatile nitrogen compounds, biological amines, carbon dioxide (CO_2_), ATP degradation products, and sulfides are commonly used for assessing the freshness of food [1]. FIs are widely used for fresh foods, including fruits and seafood.

#### 2.1.3. Leak Indicators (LI)

It is necessary to fix the internal gas concentration levels of the package to extend the shelf life of food, but the internal gas could be changed for food respiration, produced by the metabolism of spoilage microorganisms, or permeated by the packaging materials or because of package leakage (Figure 2C) [4]. Gas indicators provide an alternative method for monitoring food quality and safety by directly contacting the gases generated during food deterioration and by monitoring changes in a package’s gas composition. Now, oxygen and CO_2_ are widely used for gas indicator detection. In addition, water vapor, ethanol, hydrogen sulfide, and other gases are also used for monitoring purposes [28].

##### 2.1.4. pH Indicators (PHI)

pH indicators are another type of indicator which were developed for detecting pH value changes in packaged foods (Figure 2D) [4]. Polyphenols such as anthocyanins are currently used to develop pH indicators [27].

### 2.2. Indicator Preparation and Application

#### 2.2.1. Time–Temperature Indicators (TTIs)

TTIs are based on time and temperature to express irreversible mechanical, chemical, enzymatic, or microbial alterations of food through mechanical deformations and color changes [27]. The types of chemical or physical TTIs correspond to mechanical reactions or physical changes that occur with changes in time and temperature—for instance, acid–base reactions, melting, polymerization, etc. Some of the time–temperature indicators currently used commercially draw on physical or chemical responses, such as Monitor Mark, a typical physical and chemical TTI made on the principle that, as the temperature above the ester melting point increases, the ester diffuses more rapidly. Monitor Mark could be used to monitor a wide range of frozen or chilled foods, but it needs to be stored below the ester’s melting point before use [10].

**Figure 3:**
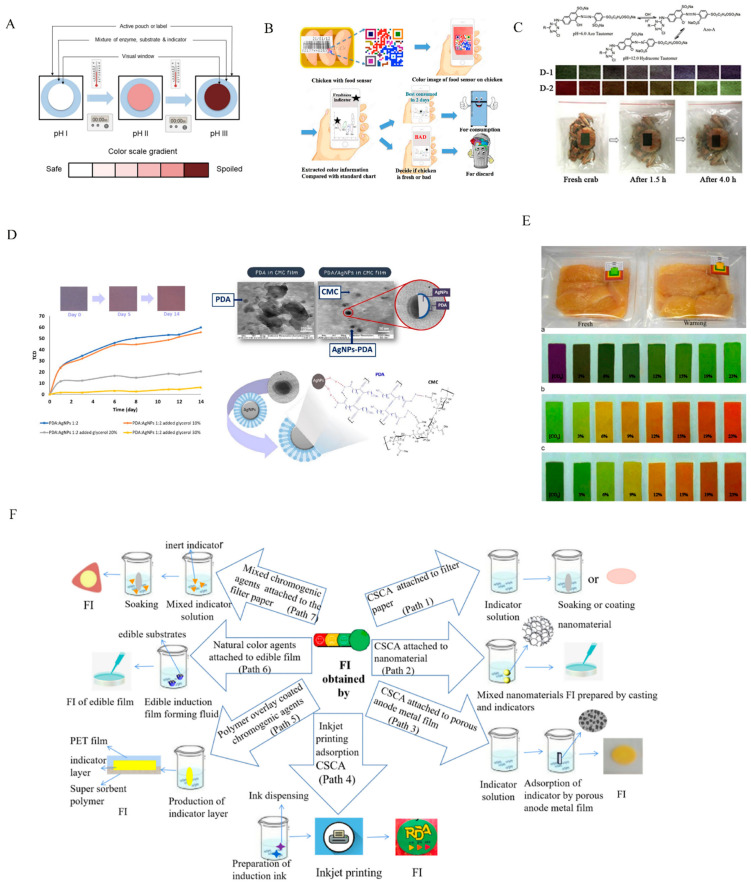
Several typical TTI and FI practical applications. (**A**) Working principle of enzyme time–temperature indicator in food packaging [11]. (**B**) Schematic of device application as food quality sensor [19]. (**C**) Intelligent color-changing paper with pH-sensitive chromophore packaging sensor [14]. (**D**) Using polydiacetylene/silver nanoparticles embedded in carboxymethyl cellulose [13]. (**E**) The food spoilage indicator that monitors the freshness of chicken breasts [16]. (**F**) Method of making FIs [2].

Biological reactions are based on changes in biological activity for the response of microorganisms, spores, or enzymes to time and temperature. The change rate is time-dependent, and changes occur more rapidly at high temperatures, similar to the food spoilage response. As shown in Figure 3A, in CheckPoint^®^ TTI, the appropriate pH-sensitive dye would change the color of the label from green to yellow to orange. The green symbol on the label indicates TTI activation, yellow indicates that the product is about to expire, and orange-red indicates that the food has expired. Appropriate changes in enzyme and substrate concentrations could control the shelf life and EA of TTIs [11]. In addition, a versatile microbial TTI was prepared by inoculating *Janthinobacterium sp*. onto tryptic soy agar with 1% glycerol. Based on the estimated ETTI and tTTI, the reference endpoint parameters, the optimal combinations of pH, initial concentration, and field volume could be determined to monitor the effect of time–temperature profiles on spoilage occurrence for different foods [12].

Photosensitive TTIs were primarily designed based on the principle that the color will change after a special photosensitive dye has been sensitized to light [29]. Saenjaiban changed the concentration ratio of silver nanoparticles (AgNPs) and glycerol to carboxymethyl cellulose (CMC), and found that the prepared TTI (AgNPs (30 mg/L) and PDA/AgNP ratio (1:3)) was sensitive to reaction (Figure 3D) [13].

The PDA/AgNP-embedded CMC films prepared by 30 mg/L AgNPs changed from violet-blue to purple and from purple to red-purple with increasing temperature [13]. In general, TTIs are widely used to establish, monitor, and evaluate changes in the shelf-life quality of various refrigerated and frozen foods due to their simplicity, low cost, and high efficiency with many commercialized TTIs in the USA (e.g., 3MTM, Fresh-Check^®^, Timestrip^®^, VarioSens^®^) [10].

#### 2.2.2. Freshness Indicators (FIs)

Freshness indicators reflect food freshness by detecting the characteristic release of food spoilage microorganisms [2]. In different types of intelligent food packaging, the main substances which could provide an indication of food freshness are CO_2_, volatile alkaline nitrogen, hydrogen sulfide, and other substances. According to the measurement results, FIs can be divided into indicators of chromogenic agents (chemical, natural) and indicators of data carriers [2]. Figure 3F outlines the method to make intelligent packaging FIs (CSCA attached to filter paper, CSCA attached to nanomaterials, CSCA attached to porous anode metal film, inkjet printing adsorption CSCA, polymer overlay coated chromogenic agents, natural color agents attached to edible film, mixed chromogenic agents attached to the filter paper).

The chromogenic agent has sufficient sensitivity within a wide pH range, and can easily distinguish the transition color when the freshness of the food changes, improving the sensitivity of the indicator [30]. The anthraquinone structure was added to the azo chromophore to expand the visible absorbing wavelength (Figure 3C). This indicator was applied to packaged cooked fresh crab, and it found that crab started to deteriorate after 1.5 h and deteriorated entirely after 4 h [14].

Polyaniline (PANI) is often used as a color developer for a fish freshness index because of its obvious color development reaction, but the sensitivity and color change range of PANI are limited, as it is not easily soluble in organic solvents. Compared with PANI samples, PANI/PSS colloids have better pH stability. Meanwhile, PANI/PSS is used to quantify the amine content in liquids and gases, and thus has good market prospects to monitor seafood freshness [15]. In addition, Lee used bromocresol blue-phenol red as an indicator for fish product freshness-indicating labels which changed color (from yellow to purple) as the pH increased [17].

Considering that it is important to distinguish the color transition in the color change process by a single indicator, a mixed indicator is proposed to improve the sensitivity of the endpoint color change. Mixed indicators can be divided into two categories: (1) adding inert dyes to acid–base indicators (color does not change at pH < 7.0); and (2) using the complementary color effects of two acid–base indicators to make the color change of the indicator more intense and narrower [2]. For meat products, two key freshness indexes are TVBN and CO_2_. A mixed indicator smart label prepared from bromocresol blue and methyl red was sensitive to CO_2_ (Figure 3E), and was used to measure chicken freshness [16].

Information about product quality, microbiological activity, or other freshness characteristics can be shown in the data carrier freshness indicators, which can be subdivided into sensors, barcodes, and radio frequency identification (RFID) according to their mode of operation [2]. Currently, commercially flexible biosensors such as Scheelite Technologies have been developed for testing *E.coli* and *Salmonella* within the food supply chain. In addition, Devi’s research found that freshness sensors developed by combining nano-metal ion polymers with xanthine oxidase were also applied for the detection of fish freshness [18]. Barcode and RFID indicators are widely used for food traceability, cost-saving, improving product quality, and controlling safety, with large-scale data storage capacity and the contactless, quick, and automatic identification of multiple products. Induction-prepared using adhesive beads and paper trays, ink-printed barcodes (colorimetric indicator arrays) can monitor and record information in real time during the food supply, are applied to monitor the spoilage process of chicken meat at 5 °C and −5 °C. The barcode (Figure 3B) can be scanned with a smartphone to obtain color information to quantitatively estimate the degree of muscle spoilage [19]. RFID is often integrated with freshness sensors to extend the functionality of RFID technology in intelligent packaging, usually by measuring relative humidity and temperature to monitor frozen fish freshness in the cold chain [2].

#### 2.2.3. Leak Indicators (LI)

**Figure 4:**
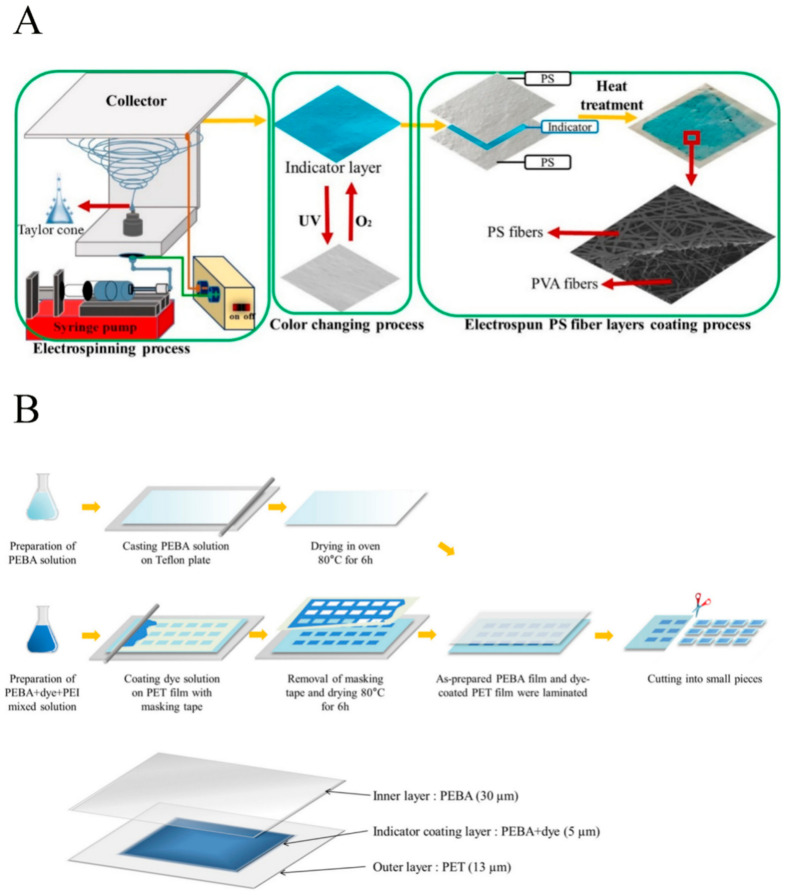
Gas indicator working principle. (**A**) Schematic drawing of 3D-printed preparation for an oxygen indicator, with the UV and oxygen causing the PS fiber layers to change color [21]. (**B**) CO_2_ indicator structure and related manufacturing process [31].

The respiration of food, the nature of packaging materials, and changes in environmental circumstances might cause changes in the composition of food packaging gases, which is directly associated with the integrity, durability, quality, and safety of packaging systems for food [4]. Oxygen indicators are widely used in food packaging to detect leaks in the packaging [10]. An oxygen indicator (UV activation method by using alginate as a coating polymer) was designed to measure the reduction of thionine leakage [20]. The oxygen indicator prepared by covering the electrospun polystyrene fiber layer with an optimized polyvinyl alcohol (PVOH) nanofiber base, with the help of 3D-printing technology, is shown in Figure 4A. The color development time of the oxygen indicator without the PS coating was found to be 1.8–189.4 s, with a leakage rate of 1.85–29.6% in the control experiment. The color recovery time of the electrospun PS fiber layer for the oxygen indicator was 38.2–240 s, and the leakage rate was 0.02–0.3% [21].

In addition, the change of the oxygen concentration of packaged food can lead to poor quality, while some of the internal microbes of food can be corrupted by changes in the CO_2_ concentration. Therefore, it is also possible that CO_2_ indicators could work as leak indicators. Similar to oxygen indicators, lysine, ԑ-polylysine, and natural anthocyanins were used to design a colorimetric CO_2_ indicator in natural extracts to determine pH changes. In poultry meat experiments, water-type and label-type indicators were investigated at various CO_2_ levels, and a color change from sky blue to dark purple was observed [7]. Indicators prepared according to the PET/PEBA + dye (MR + BTB) (3:7) + PEI 5%/PEBA combination ratio were sensitive to changes in CO_2_ concentration (Figure 4B) [31].

#### 2.2.4. PH Indicators (PHI)

**Figure 5:**
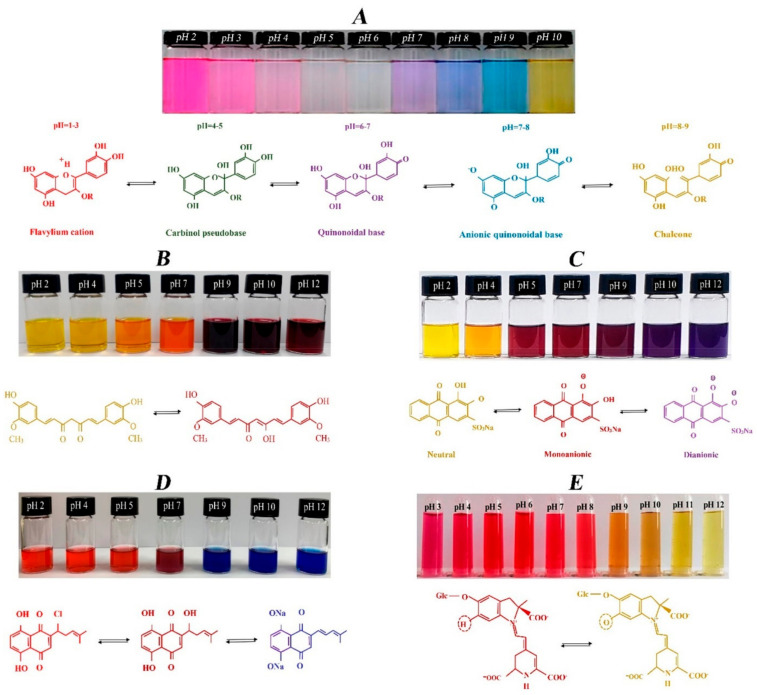
The color changes of (**A**) anthocyanin, (**B**) curcumin, (**C**) alizarin, (**D**) shikonin, and (**E**) betalains at different pH levels [3].

Curcumin, alizarin, shikonin, betalains, and other pigments are widely available, are sensitive to pH changes, and have a wide response range, which affords them good application value [3]. The differences in the color development of several natural pigments under various pH conditions are displayed in Figure 5. At present, pH indicators are mainly prepared with other materials compounded according to the natural pigments’ high pH sensitivity.

**Figure 6:**
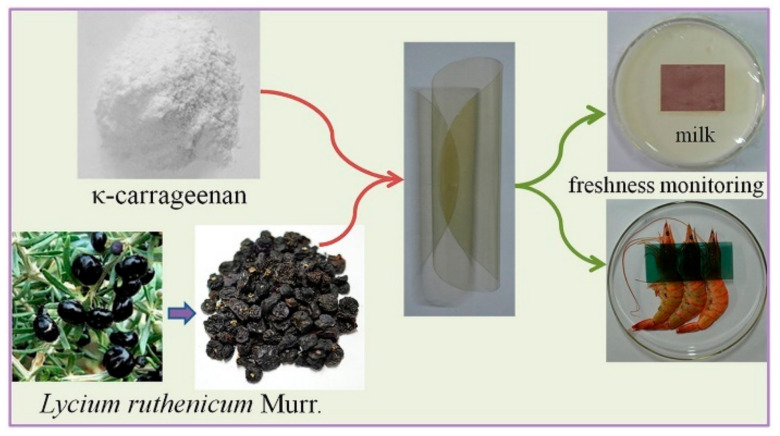
Applications of colorimetric film for milk and shrimp freshness monitoring [24].

A colorimetric pH indicator was prepared by using potato starch as an immobilizer for the natural pigment anthocyanin. An obvious change from red to green was found between the pH and the spoilage of the meat, while fresh pork samples had a pH value of 5.18–6.16 [22]. Using the antioxidant and antimicrobial properties of these natural pigments, these pH indicators showed versatile applications [4,32]. In addition, a colorimetric film prepared from κ-carrageenan and black fruit wolfberry is presented in Figure 6; the indicator’s color changed reversibly from pH 2 to 10, from pink to colorless in the range of pH 2 to 6, and from blue-violet to yellow in the range of pH 7 to 10. The film can also indicate fish product freshness [2].

## 3. 3D-Printing Technology and Intelligent Packaging

### 3.1. Introduction of 3D Printing

The technology of 3D printing has been used extensively in the food processing field for economic and environmental benefits, not only enabling industrial mass production for complete automation, but also creating customized classes of food according to the different needs of consumers and sufficiently complex structures [33,34].

Essentially, 3D-printing technology provides an engineering solution for the development of personalized custom foods and facilitates the development of food products, and can also reconfigure the machines of the custom food supply chain [35]. In addition, 3D-printing technology makes the real-time detection of meat freshness/degradation possible; the freshness of meat can be determined by testing the pH. Therefore, we could prepare composite materials using substances which are sensitive to pH and could be indicative of this change (e.g., anthocyanins). The application of 3D-printing technology to printing food packaging film can not only achieve rapid results in terms of checking the freshness of meat, but can also extend the shelf-life of food by insulating it from air through a food packaging film.

### 3.2. 3D-Printing Technology for Indicators

As an attractive an emerging technology in the food sector, 3D printing has strong advantages. As an extension of 3D printing, combining 3D printing with indicators is a very creative initiative, similar to the 4D-printing concept. The technology of 3D printing could transform the physical and chemical properties of food into a macroscopic color change by adding substances, which change color due to external stimuli, making it easier for consumers to observe the freshness/perishability of food. The color-changing properties of anthocyanins have been used in food packaging films’ production to monitor pH changes, while their antioxidant activity has been proven to be beneficial to human health [36].

**Figure 7:**
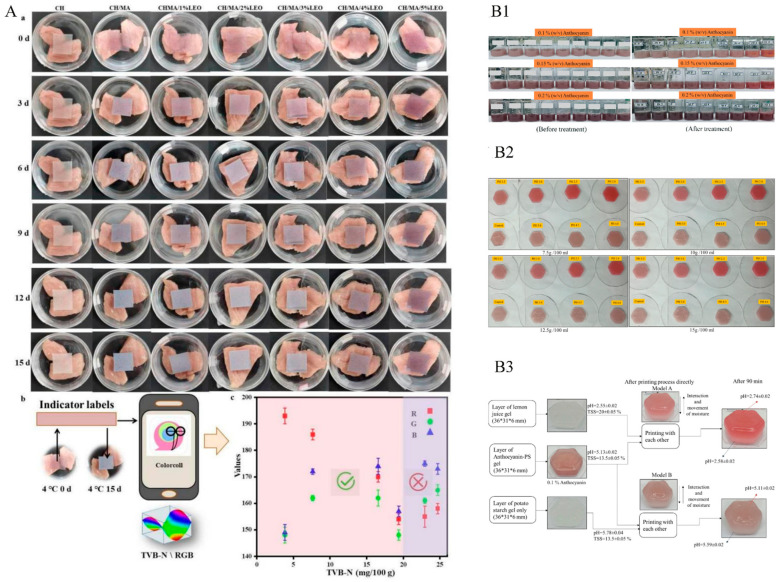
The application of 3D printing in intelligent packaging. (**A**) The 3D printing of MA as a bioindicator film for dyes [37]. (**a**) Pictures of the indicator film stored at 4 °C; (**b**) schematic diagram of the method for assessing meat quality; (**c**) TVB-N results and RGB data for assessing the freshness of pork. (**B**) Principle of applying anthocyanin-P°CS gels for the 3D printing of intelligent food packaging indicators [38]; (**B1**) color of anthocyanin–PS gels before and after treatment (pH 2–10); (**B2**) anthocyanin–PS gels color change (pH 2 to 5 and PS 12.5–15%); (**B3**) design of the 3D-printed model.

Li et al. adopted a method that used chitosan (CH), mulberry anthocyanin (MA), and lemongrass essential oil (LEO) as biocomposites, and cassava starch (CS) as an indicator film to form a protective layer [37]. This film not only showed the LEO’s antioxidant and antimicrobial properties, but also used the LEO’s sensitivity advantage of temperature and pH to show different color changes (Figure 7B) to detect the freshness of pork. Ghazal used anthocyanin–potato starch gels as inks for 3D printing [38]. The effects of different pH and concentration ratios of anthocyanin-PS on gels are shown in Figure 7(B1) and Figure 7(B2), respectively. The results of this study could also be applied as a new pH indicator (Figure 7(B3)), leading to the development of additive manufacturing in the food industry.

Notably, 3D-printed food packaging films using composite materials could be used to package fish products [39]. Considering fish product problems such as spoilage, parasites, and dehydration, it would be possible to consider adding antibacterial substances or water-retaining ingredients as composites to make the product more marketable. In addition, the food packaging films produced by 3D-printing technology with chitosan not only demonstrated better antibacterial properties compared to the control films, but also had better elasticity and elongation, so more substances suitable for use in the preparation of biocomposites are waiting to be exploited by future researchers [40,41]. Generally, the cost of 3D printing is still challenging for large-scale applications, especially in low-profit fields. However, the current development of novel techniques in 3D printing supports new possibilities for intelligent food packaging.

As above, intelligent food packaging is not only a major trend, but also an important safeguard for food safety which can easily protect some perishable food, such as fresh fruits, vegetables, seafood, meat, etc. The future trend of the food market would lead to greener, safer, and more intuitive and advanced intelligent food packaging that could record and transmit real-time data and achieve food traceability through smart devices. Such 3D-printing technologies have extensive usage in the food industry, and the combination of 3D-printing technology and indicators could be used increasingly widely in the food sector in the future, which would promise a direction for future food research and development.

## 4. Challenges and Outlook

**Figure 8:**
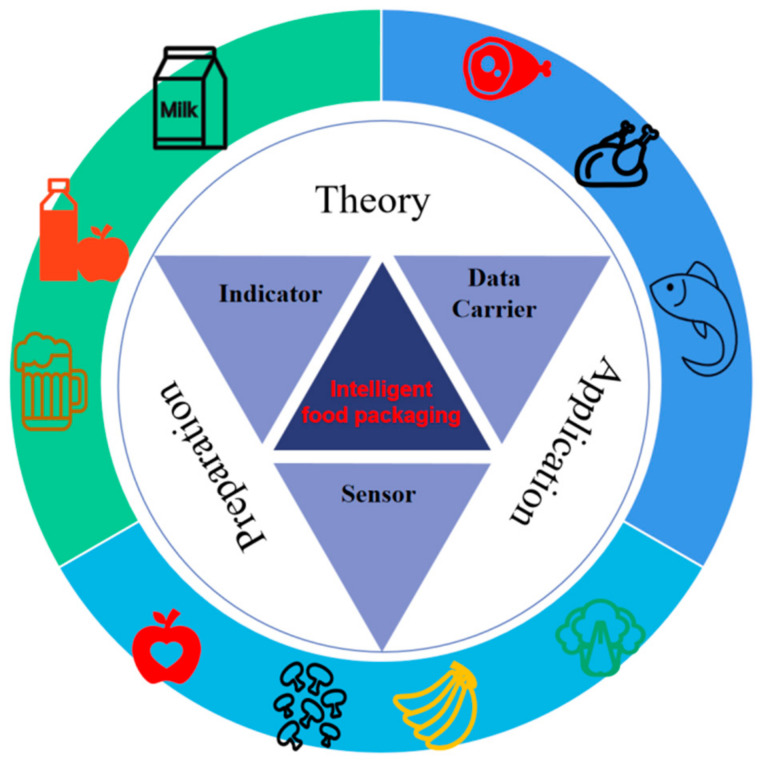
Framework of intelligent food packaging indicators for monitoring food spoilage.

Intelligent food packaging does not only protect and contain the food, compared to traditional food packaging, but is combined with modern technologies as a way of transmitting information. In the future, there is a great deal of scope for the advancement of intelligent indicator-based packaging technology in the food sector (Figure 8). Indicators provide visualizations of food freshness, real-time and continuous monitoring of the status of food, and effectively improve food safety and reduce food waste and spoilage.

Mixed colorants expand the range of colors, compared to single colorants, so that the freshness of food can be detected clearly. Consumers can use electronic devices such as mobile phones to monitor the freshness of food in real time, and can use electronic devices to obtain more intuitive and precise data. Because general non-contact indicators are usually attached to the packaging interior, and only some volatile substances can be detected, researchers can try to develop contact-type indicators that can detect substances on food surfaces. Obviously, an inevitable trend for indicators is the pursuit of safe, green, and sustainable intelligent food packaging.

It was considered that the composition is mainly a natural polymer that cannot be precisely controlled by various mechanical parameters. Because intelligent food packaging film can make direct contact with food, it is necessary to further evaluate the safety, including compounds’ migration, which makes it difficult to achieve industrial applications. It is worth noting that the sensitivity of indicators may be affected by the packaging conditions of different foods, which may affect consumers’ judgment of food quality. Research and development of intelligent food packaging is still forthcoming, as the regulations for food safety in various countries are different, which greatly increases the cost of intelligent food packaging.

Intelligent food packaging is a decisive part of future food packaging development. The development of natural coloring agents is the focus of future researchers, because the application of food products to ensure the safety and non-toxicity of the substrate, to avoid the migration of compounds, and to improve the stability of indicator, are the main directions of subsequent developments of this technology. Combining 3D-printing technology with indicators has created a scientifically customized packaging system that not only satisfies food safety and sustainable green development, but also prompts the food sector to explore major changes to the optimal taste period/expiration date. As an extension of traditional food packaging, intelligent food packaging created by the application of 3D-printing technology realizes real-time, low-cost, and continuous food monitoring.

## Figures and Tables

**Table 1:** Classification and application of indicators in food packaging.

Name	Type	Introduction	Food	Precursors	Performance	Ref
Monitor Mark	TTI	Indicators prepared according to the melting point of lipids, which were sensitive to temperature changes and needed to be stored at a temperature below the melting point of the ester.	Frozen or refrigerated food products.	Polyester film layers.	Discoloration of holes on TTI.	[10]
CheckPoint^®^ TTIs		CheckPoint^®^ displays different colors at different pH substrate values, ranging from green to yellow to orange-red.	Fruit and meat products.	Lipases, lipid-backed aqueous solutions, and acid–base indicators.	Color changes.	[11]
Microbial TTIs		Janthinobacterium sp. medium was sensitive to NaCl concentration changes, and experimental results found that NaCl concentration was proportional to the detection range of the sensor.	Fresh and ground pork meat, meat products.	1% glycerol, spot-inoculated.	Color change of the spots.	[12]
AgNPs-TTIs		AgNPs damage the structure of PDA to increase the mobility and surface area of PDA. The ADA/AgNP bilayer structure was inhibited by glycerol. Embedding PDA/AgNPs into CMC films can be used as TTI films for fruits and vegetables.	Fresh apples, whole carrots, lettuce, strawberries, and mushrooms.	Silver nanoparticles (AgNPs) and glycerol.	Color change of PDA/AgNPs.	[13]
D-1, D-2	FI	In 6.0–12.0, the D-1 samples ranged from green to purple, and in 1.0–4.0, the D-2 samples ranged from red to green. The color variation of the imprinted pattern was obvious, varied, and easy to distinguish.	Crab.	Sensitive reactive dyes.	Color change of the paper printing.	[14]
PANI/PSS		When TVB-N was close to the critical value, the polyaniline film, washed with HCl, still showed obvious color changes from green to peacock blue, and could be recycled at least three times.	Tilapia.	A renewable indicator based on polyaniline (PANI).	Color change of PANI.	[15]
Bromothymol blue and methyl red (M2 type)		Bromocresol green, changing from alkaline form (blue-green, pH 5.4) to acidic form (yellow, pH 3.8) after being exposed to CO_2_. The mixed dye-type indicator absorption peak shifted from 558–562 nm to 430–435 nm.	Chicken.	A colorimetric mixed-pH dye-based indicator.	Total color difference of a mixed-pH dye-based indicator.	[16]
BTB-PR		The correlation coefficient between the pH and ammonia concentration was found to be r2 = 0.9866. The indicator label changed from yellow to purple after longer storage time.	Raja kenojei.	Consisting of the pH-sensitive dye, bromothymol blue-phenol red (BTB-PR).	The pH values of skate and the chromaticity of the gas indicator were measured.	[17]
XOD/CHIT/Fe-NPs@Au/PGE		The biosensor operated in a range from 0.1 to 300 lM, a detection limit of 0.1 lM (S/N = 3), 0.001169 mAlμM^−1^cm^−2^ sensitivity, 0.99 correlation coefficient, and a rapid response (<3 s) of 0.5 V at one potential. At the same time, the stability was higher.	Fish.	A xanthine biosensor was fabricated using XOD/CHIT/Fe-NPs@Au/PGE as the working, Ag/AgCl as the reference, and Pt as the auxiliary electrode.	The sensitivity of the biosensor.	[18]
Paper-based colorimetric sensor arrays		A smartphone was used to read the sensor information and found that the same cross-reactive pH and VOC-sensitive dyes could be used to monitor the aging of chickens to some extent. Temperature-aging of food products was very responsive to storage temperature changes. When the meat degrades at a faster rate, the color curve changes the most.	Chicken.	The food’s barcode.	Color information.	[19]
Water-resistant UV-activated oxygen indicator	LI	Alginate was used as the coating polymer to prepare UV–oxygen indicator films. When the concentration of alginate increased to 1.25%, the dye leaching rate was significantly reduced to 5.80 0.06%.	Food.	The dye-based oxygen indicator film.	Suffers from dye leaching upon contact with water.	[20]
PVOH nanofiber-based oxygen leakage indicators		The indicator light interacted with oxygen and turned blue. The color of the uncoated indicator returned to a lower color than the original. This could be attributed to an increasing relative humidity in the package during the 10-day storage period.	Meatballs.	Covering the optimized polyvinyl alcohol (PVOH) nanofiber-based oxygen indicator with electrospun polystyrene (PS) fiber layers.	Significant color changes.	[21]
Lysine/poly-lysine/anthocyanins–CO2 indicators		Indicator still detects food freshness at 0.01% *w*/*v* concentration. Color changes are also observed in the 0.1% *w*/*v* solution when CO_2_ concentration decreased below 20%. Dissolved in the biopolymer matrix at this point, it forms a coating consisting entirely of food-grade components that can be used as a colorimetric CO_2_ indicator for refrigerated foods.	Poultry meat.	An amino acid (L-Lysine), a polypeptide (ε-poly-l-lysine, EPL), and natural occurring dyes (anthocyanins).	Color changes.	[7]
Colorimetric pH indicator film	PHI	Immobilization of natural dye anthocyanins was based on AGAR and potato starch. The extracts of AGAR, starch, and anthocyanin were compatible.	Pork.	A new colorimetric pH indicator film.	The color changes.	[22]
CS-TO2 and CS-TiO2-BPPE film		Cs-TiO_2_-bppe film has antioxidant, ethylene-scavenging, and antibacterial properties. These films are PH sensitive.	Fish.	Films with chitosan (CS), 9 nanometer TiO_2_, and black plum peel extract (BPPE) as the main raw materials.	Free radical scavenging activity.	[23]
κ-carrageenan incorporated with Lycium ruthenicum film.		The film’s thermal stability and water vapor barrier properties were improved to some extent when the LRM incorporation was low (not more than 2.5%). The films exhibited color in the pH range of 2 to 10, while the color change was reversible and had good antioxidant activity.	Aquatic products.	A novel, wide pH-sensing colorimetric film.	The color changes.	[24]

## Data Availability

Not applicable.
